# Deforestation Increases Developmental Instability in the Orchid Bee *Euglossa imperialis* Across Protected Areas of Panama

**DOI:** 10.3390/insects17080818

**Published:** 2026-08-06

**Authors:** Fernando Moya, Tamara Segovia-Jara, Valeria Tapia, Catalina Aguilar, Jordan Hernandez-Martelo, Laura M. Pérez, Manuel J. Suazo, Alexis Matheu, Yostin Añino, Dumas Gálvez, Emilio Romero-Romero, Alonso Santos, Carmen Rodríguez-Poveda, Hugo A. Benítez

**Affiliations:** 1 Millenium Institute Biodiversity of Antarctic and Subantarctic Ecosystems (BASE), Santiago 7800003, Chile; fernando.moya@ug.uchile.cl (F.M.); catalina.aguilar@ug.uchile.cl (C.A.); jordan.hernandez.01@alumnos.ucm.cl (J.H.-M.); 2 Laboratorio de Ecología Molecular, Departamento de Ciencias Ecológicas, Facultad de Ciencias, Universidad de Chile, Santiago 7800003, Chile; 3 Laboratorio de Ecología y Morfometría Evolutiva, Instituto One Health, Facultad de Ciencias de la Vida, Universidad Andrés Bello, República 440, Santiago 8370134, Chile; tamarasegovia2001@gmail.com (T.S.-J.); valeria.tapia2017@umce.cl (V.T.); 4 Programa de Magister en Entomología, Instituto de Entomología, Universidad Metropolitana de Ciencias de la Educación, Av. José Pedro Alessandri 774, Santiago 8320000, Chile; 5 Programa de Doctorado en Salud Ecosistémica, Centro de Investigación de Estudios Avanzados del Maule, Universidad Católica del Maule, Talca 3460000, Chile; 6 Cape Horn International Center (CHIC), Centro Universitario Cabo de Hornos, Puerto Williams 6350000, Chile; 7 Departamento de Ingeniería Industrial y de Sistemas, Universidad de Tarapacá, Arica 1000000, Chile; lperez@academicos.uta.cl; 8 Vicerrectoría de Investigación y Postgrado, Universidad de La Serena, La Serena 1700000, Chile; suazo.mj@gmail.com; 9 Centro de Investigación Institucional, Universidad Bernardo O’Higgins, Santiago 8370993, Chile; alexis.matheu@ubo.cl; 10 Museo de Invertebrados G.B. Fairchild, Universidad de Panamá, Ciudad de Panamá 0824-0021, Panama; emilioromero2011@gmail.com (E.R.-R.); santosmurgasa@gmail.com (A.S.); 11 Coiba Scientific Station, City of Knowledge, Clayton 0843-01853, Panama; dgalvez@coiba.org.pa; 12 Programa Centroamericano de Maestría en Entomología, Universidad de Panamá, Ciudad de Panamá 0801, Panama; 13 Sistema Nacional de Investigación (SNI), Ciudad de Panamá 0824-0021, Panama; 14 Departamento de Matemática, Universidad de Panamá, Panamá City 0801, Panama; carmen.rodriguezp@up.ac.pa; 15 Centro de Investigación de Estudios Avanzados del Maule, Universidad Católica del Maule, Talca 3466706, Chile

**Keywords:** fluctuating asymmetry, developmental instability, Euglossini, habitat degradation, wing shape, geometric morphometrics

## Abstract

Habitat loss and deforestation are among the main drivers of biodiversity decline in tropical ecosystems, yet their effects on developmental stability remain poorly understood in many insect groups. Orchid bees (Apidae: Euglossini) have been proposed as potential bioindicators of environmental quality due to their close association with forested habitats and sensitivity to habitat disturbance. Here, we evaluated the relationship between forest cover, wing morphology, and developmental stability in the orchid bee *Euglossa imperialis* across five protected areas of Panama exhibiting different levels of deforestation. A total of 290 individuals were analyzed using geometric morphometrics and fluctuating asymmetry (FA) of the forewing. Morphological variation was assessed through Principal Component and Canonical Variate Analyses, whereas developmental instability was quantified using Procrustes ANOVA and FA scores. Wing shape showed moderate differentiation among sites, with La Tronosa, the most deforested protected area, exhibiting the greatest morphological divergence. In contrast, FA displayed a clear association with habitat degradation. La Tronosa presented the highest levels of developmental instability, and generalized linear models revealed a significant positive relationship between deforestation and FA at both local and landscape scales, with landscape-level deforestation showing the strongest effect. Our results indicate that developmental instability is more sensitive than overall wing morphology for detecting the biological consequences of habitat alteration. These findings support the use of fluctuating asymmetry as an early indicator of environmental degradation and provide new evidence for the value of orchid bees as bioindicators in tropical ecosystems.

## Simple Summary

Tropical deforestation is one of the main threats to biodiversity, but its effects on insect development are still poorly understood. Orchid bees are important pollinators that depend on forest habitats and may serve as indicators of environmental quality. We studied populations of the orchid bee *Euglossa imperialis* from five protected areas in Panama with different levels of forest loss. We found that bees from the most deforested areas showed greater developmental instability, measured through small differences between the left and right wings, while overall wing shape changed only moderately among sites. Developmental instability increased with increasing deforestation, particularly at the landscape scale. These results suggest that fluctuating asymmetry is a sensitive indicator of habitat degradation and reinforce the value of orchid bees as bioindicators for monitoring the impacts of deforestation in tropical ecosystems.

## 1. Introduction

One of the most consistent patterns in ecology is the latitudinal gradient of biodiversity, whereby species richness generally increases toward the equator and reaches its highest values in tropical regions [1,2]. Although this pattern applies to many insect groups, important exceptions have been documented. Bees, for example, often exhibit peak diversity in subtropical or temperate regions, whereas orchid bees (Euglossini) and stingless bees (Meliponini) follow the more typical tropical diversity pattern. Likewise, some hymenopteran groups such as sawflies do not conform to the classical latitudinal diversity gradient [3,4]. The classical latitudinal diversity gradient is particularly evident in insects, which represent the most diverse and abundant group of terrestrial organisms and play essential roles in pollination, nutrient cycling, decomposition, and trophic interactions [5]. Despite their ecological importance, increasing evidence suggests that insect populations have undergone significant declines in recent decades, raising concerns about the conservation of tropical biodiversity and the maintenance of ecosystem functioning [6].

Among the multiple drivers proposed to explain insect declines, habitat loss and deforestation are considered among the most pervasive threats to tropical biodiversity [7,8]. The conversion of natural habitats into agricultural, urban, and other human-modified landscapes alters environmental conditions, reduces resource availability, and disrupts ecological interactions that sustain insect communities [9]. In addition to habitat degradation, other anthropogenic pressures such as pollution, biological invasions, and climate change further contribute to biodiversity loss and ecosystem alteration [10]. As a consequence, understanding how organisms respond to habitat transformation has become a central challenge for biodiversity conservation and ecological monitoring.

One of the most informative approaches for evaluating biological responses to environmental change is the study of morphology. Morphological traits often reflect the interaction between genetic, developmental, and environmental factors, providing valuable insights into how organisms respond to habitat alteration and ecological stress [11]. In insects, wing morphology has proven particularly informative because it is closely associated with dispersal, flight performance, and reproductive success. Geographic and climatic variation, including temperature and precipitation, has been associated with changes in wing size and shape in several insect groups [12,13]. Chemical exposure and other anthropogenic stressors can also produce detectable changes in wing morphology and developmental stability [14,15]. However, these responses are not universal and may vary according to species, sex, developmental stage, the morphological trait examined, and the intensity or duration of environmental exposure [16]. Consequently, quantifying morphological variation can contribute to understanding ecological adaptation, habitat use, and the mechanisms underlying species responses to environmental disturbance [17,18].

Beyond overall morphological variation, environmental stress can also affect developmental stability, defined as the ability of an organism to produce a consistent phenotype despite genetic and environmental perturbations during development [19,20]. When developmental buffering mechanisms are compromised, small random deviations may accumulate, generating developmental instability that can be detected through fluctuating asymmetry (FA), a widely used indicator of biological stress and environmental disturbance [21,22,23]. Because FA reflects subtle developmental disruptions before major morphological changes become evident, it has become a valuable tool in studies of conservation biology, environmental monitoring, and organismal responses to habitat alteration [24,25].

To assess biodiversity status, ecosystem health, and the consequences of environmental change, reliable bioindicators are required [26]. Bioindicators are organisms whose responses to environmental perturbations can be used to infer ecosystem condition, providing valuable tools for ecological monitoring and conservation [27,28,29,30]. Because they respond to environmental variation at behavioral, physiological, molecular, and morphological levels, insects are among the most widely used bioindicator groups due to their high diversity, abundance, and sensitivity to environmental disturbances [31,32,33].

Among insects, orchid bees (Hymenoptera: Apidae: Euglossini) have emerged as a valuable model for studying the ecological consequences of land-use change [34,35]. This group comprises approximately 200 species distributed throughout the Neotropics, with its highest diversity concentrated in tropical forests [4,36,37,38,39]. Orchid bees are highly associated with forested habitats and are particularly sensitive to habitat disturbance, with only a subset of species persisting in altered landscapes [39,40,41]. These characteristics have led to their proposal as potential bioindicators of environmental quality and conservation status [42].

Despite their potential, the usefulness of orchid bees as bioindicators remains under debate. Añino et al. [43] questioned whether Euglossini consistently fulfill the criteria required of effective bioindicators, particularly regarding their responses to environmental change. In contrast, Gonçalves and Faria [44] argued that orchid bees possess several ecological attributes that support their use in conservation monitoring, including their close association with forest habitats, sensitivity to habitat disturbance, relatively well-known taxonomy, ease of standardized sampling using scent-baited traps, and their important role as pollinators in Neotropical ecosystems. This debate highlights the need for additional approaches capable of evaluating how habitat alteration affects orchid bee populations and determining their suitability as indicators of environmental degradation.

Despite the growing interest in orchid bees as indicators of environmental quality, relatively little is known about how habitat degradation influences their developmental stability and morphological responses. Because environmental stress can affect developmental processes before producing detectable changes in abundance or community composition, evaluating developmental instability may provide a sensitive approach for detecting the biological consequences of habitat alteration. In this context, geometric morphometrics offers a powerful framework for quantifying subtle patterns of morphological variation, while fluctuating asymmetry provides an indicator of developmental stress and instability [16,45]. Together, these approaches offer an opportunity to assess whether deforestation generates measurable biological responses in orchid bee populations and, consequently, whether these responses can contribute to their use as indicators of environmental degradation.

Therefore, this study evaluated the relationship between forest cover, deforestation, wing morphology, and developmental stability in *Euglossa imperialis* across five protected areas of Panama. We used fluctuating asymmetry as an indicator of developmental stress at two spatial scales: a landscape scale, comparing protected areas with different levels of forest cover, and a local scale, comparing forested and deforested sectors within each protected area. We hypothesized that individuals inhabiting more deforested landscapes would exhibit higher levels of fluctuating asymmetry, reflecting increased developmental instability associated with habitat degradation.

## 2. Materials and Methods

### 2.1. Study Site and Environment Characterization

**Figure 1:**
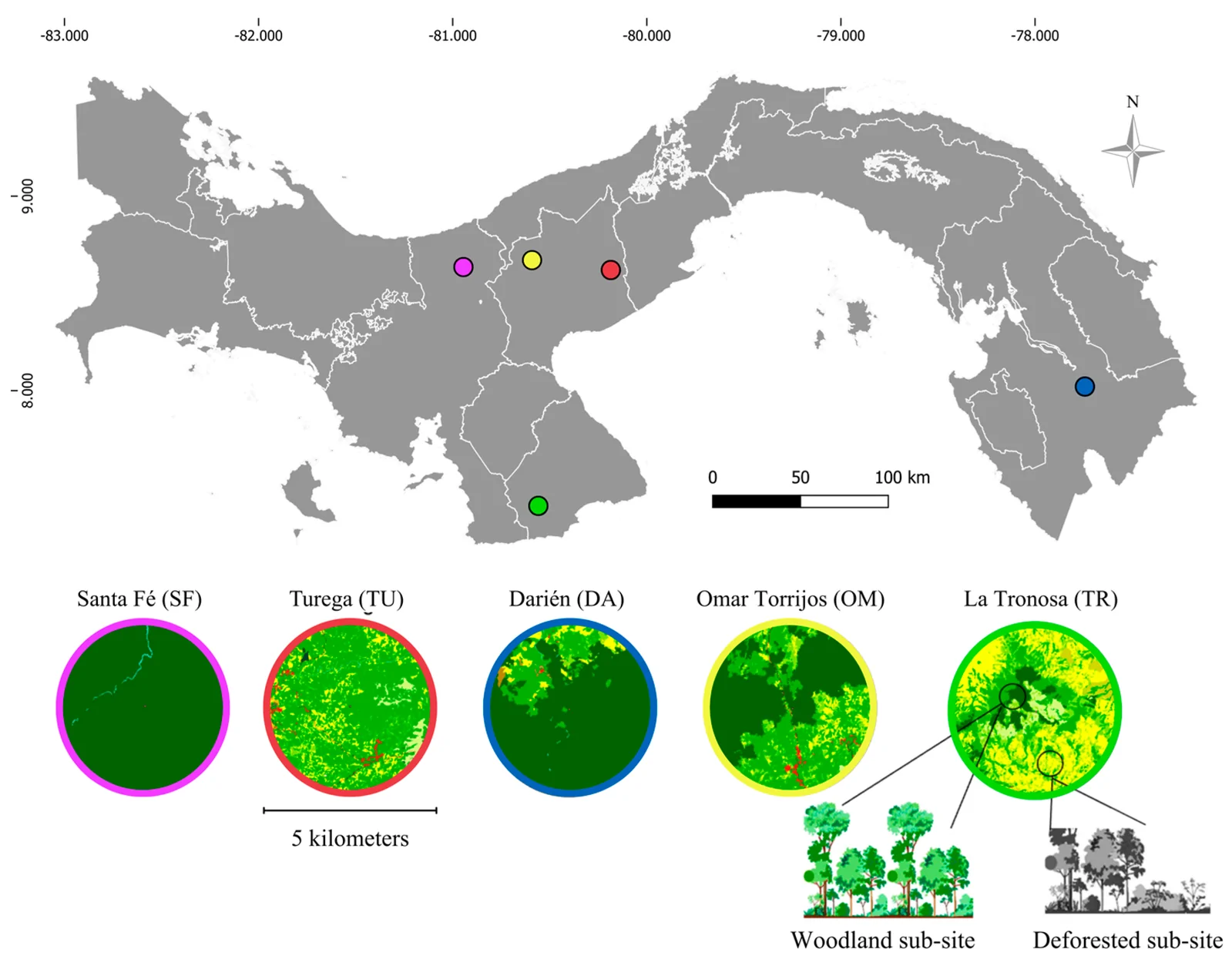
Geographic location of the five protected areas used in this study. Buffers for each site with their land cover. An example of woodland and deforested sub-sites is shown. La Tronosa (TR, green), Túrega (TU, red), Omar Torrijos (OM, yellow), Darién (DA, blue), and Santa Fé (SF, purple).

To evaluate relationships between vegetation coverage and morphology, five protected areas in Panamá were sampled (Figure 1), Darien National Park (DA; 8.01623497, −77.744262); La Tronosa Forest Reserve (TR; 7.40611, −80.55333); Omar Torrijos Herrera National Park (OT; 8.67211332, −80.592397); Santa Fé National Park (SF; 8.6347778, −80.94408); and Túrega Water Reserve (TU; 8.61917, −80.19083), all of them located in the mainland. These sites are areas with significant forest cover; within them, there are zones with forest cover and surrounding areas with varying degrees of deforestation (see Santos Murgas and Añino Ramos [35]; Santos Murgas [46]). Inside each protected area, a random point was selected and a 2.5 km radius buffer (site) was established. Within each buffer, vegetation cover was characterized using the “MiAmbiente” land-cover and land-use raster (2021 release; cartographic year 2019). The percentage of each land-cover category was quantified using geoprocessing tools in QGIS v. 3.40. To ensure that the land-cover data accurately represented conditions during the study period, all the sites were verified using drone imagery prior to field sampling, confirming that no substantial changes in vegetation cover had occurred [47]. Two contrasting land-cover variables were estimated: (I) deforested cover, representing anthropogenic land cover and including herbs, grass, stubble, and other minor land-cover categories (e.g., crops and infrastructure); and (II) woodland cover, including primary and secondary forest, representing the non-deforested landscape.

On a micro scale of evaluation, two points in each site were chosen (sub-sites) to compare morphology: one woodland sub-site and one deforested sub-site (Figure 1). The paired sub-sites were separated by an average distance of 3.34 ± 0.47 km. On a macro scale, each protected area was classified according to the percentage of woodland and deforested coverage. This was realized by classifying a coverage range of 0–40% as low level, 41–70% as medium level, and 71–100% as high level. Here, morphology was compared between the sites.

### 2.2. Sampling and Treatment of Bees

In total, 290 individuals of *E. imperialis* were collected for morphological analyses (DA, n = 60; TR, n = 57; OT, n = 55; SF, n = 58; TU, n = 60). Sampling effort was standardized by using the same number of traps, trap type, bait, and sampling duration at each protected area. The resulting sample sizes were similar among the sites, facilitating morphometric comparisons. Two visits to each site were made during January and May 2023: one to set the collection traps and the other to remove them, with an interval between setting and removing the traps of approximately 28 days. Six modified bottle traps baited with eucalyptus oil were used following Santos Murgas and Añino Ramos [35]. The captured bees were preserved in 70% ethanol and transported to the G.B. Fairchild Museum of Invertebrates (MIUP), University of Panama, where they were mounted on entomological pins and labeled. Voucher specimens were deposited in the G.B. Fairchild Museum of Invertebrates (MIUP), University of Panama. The individuals were identified using the taxonomic keys of Roubik and Hanson [4].

**Figure 2:**
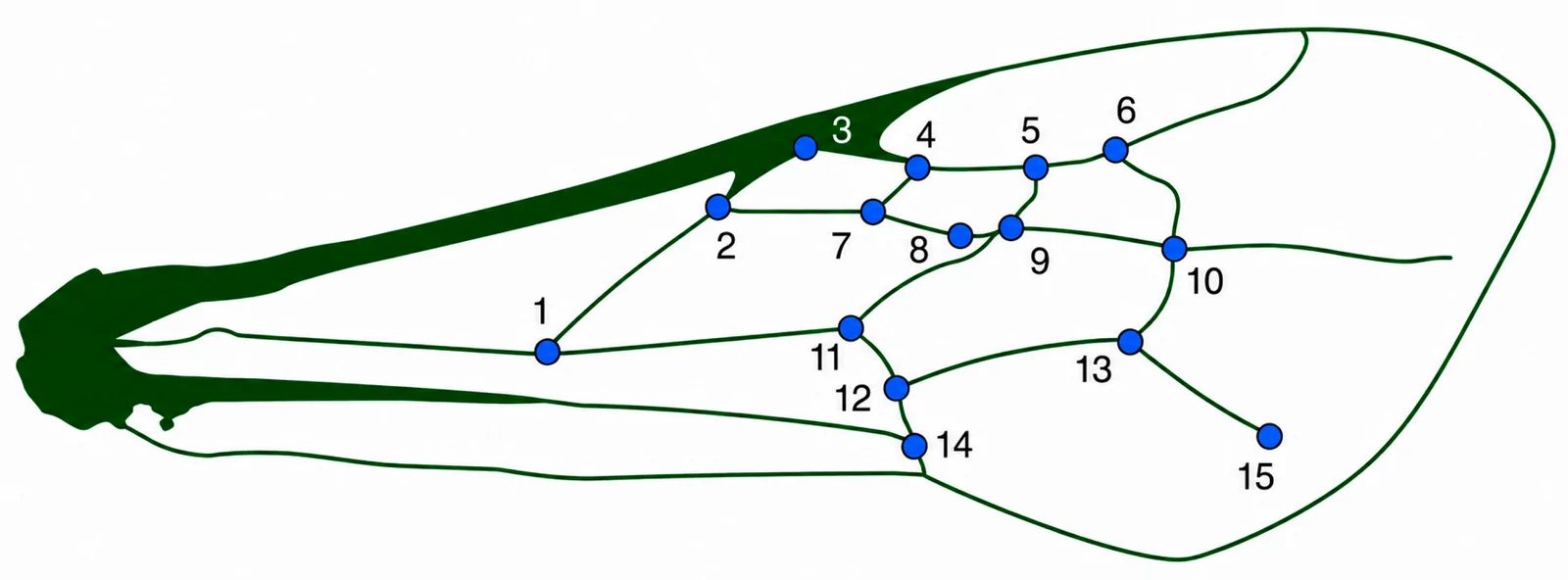
Representation of *Euglossa imperialis* forewing, showing the 15 landmarks used to characterize wing shape.

To realize the specimens’ treatment, the forewings of each *E. imperialis* individual were carefully removed, air-dried, mounted on slides, and secured with transparent adhesive tape. Each left- and right-mounted forewing was photographed using a Leica M205A stereo microscope (Wetzlar, Germany) at 4X magnification with a built-in camera and a calibrated scale. The individuals with damaged or incomplete wings were excluded [48]. The photographs were compiled in a tps format using the tpsUtil software v1.81 [49]. Fifteen landmarks were established and digitized at vein intersections (Figure 2) using tpsDig2 software v2.31 [50,51]. To estimate the measurement error and fluctuating asymmetry, landmark digitization was performed twice per individual at different times [52,53]. Morphometric information was extracted by a Procrustes Fit, which eliminates the effects of size, rotation and position [54].

### 2.3. Morphometric Analyses

To visualize the wings' morphospace and evaluate shape variations, a Principal Component Analysis (PCA) was performed using the Covariance Matrix of Procrustes coordinates [55]. In addition to this, new shape axes were generated through a Canonical Variate Analysis (CVA), allowing a reduction in the number of shape dimensions and maximizing the differentiation between the sites and sub-sites. Also, to statistically evaluate the differences in morphology between the sites and sub-sites, a permutation test with 10,000 rounds was performed using the Mahalanobis shape distances (extracted from the CVA), which represent the magnitude of difference between average shapes [56].

On the other hand, to evaluate developmental instability, fluctuating asymmetry was quantified for each site and sub-site by performing a Procrustes ANOVA, which estimates the effect of individual, side (directional asymmetry), individual side (fluctuating asymmetry) and measurement error on the asymmetric shape component [57]. To estimate whether levels of FA differ between the sites, a Kruskal–Wallis test was performed (differences in mean between multiple independent groups. Similarly, to evaluate significant differences in FA between the sub-sites, a Man-Whitney U test was performed comparing FA values between the sub-sites for each site. Finally, to determine the influence of deforestation on the FA levels, a generalized linear model with Gaussian distribution (link identity) was realized using the individual FA as a response variable, and two predictors: deforested variables (herbs + grass + stubble + others) and sub-site factors. The PCAs, CVAs, and boxplots were visualized in plots using the “ggplot2” package version 3.5.1 [58]. All the geometric morphometrics and statistical analyses were conducted through the software MorphoJ v1.07 [59] and the package “geomorph” V. 4.0.10 in R software v 4.04 (Adams et al., 2026).

## 3. Results

Quantification of the land cover revealed variations between protected areas in the proportion of forest and deforested land. Turega, Darien, Omar Torrijos and Santa Fé showed low deforestation and high forest cover. Santa Fé recorded 100% primary forest (0% deforestation), followed by Darien (2%), Omar Torrijos (20%), and Turega (21%). The remaining site, La Tronosa National Park, presents a different composition, with a medium coverage of deforested variables (50%), being the most disturbed protected area of the five.

**Figure 3:**
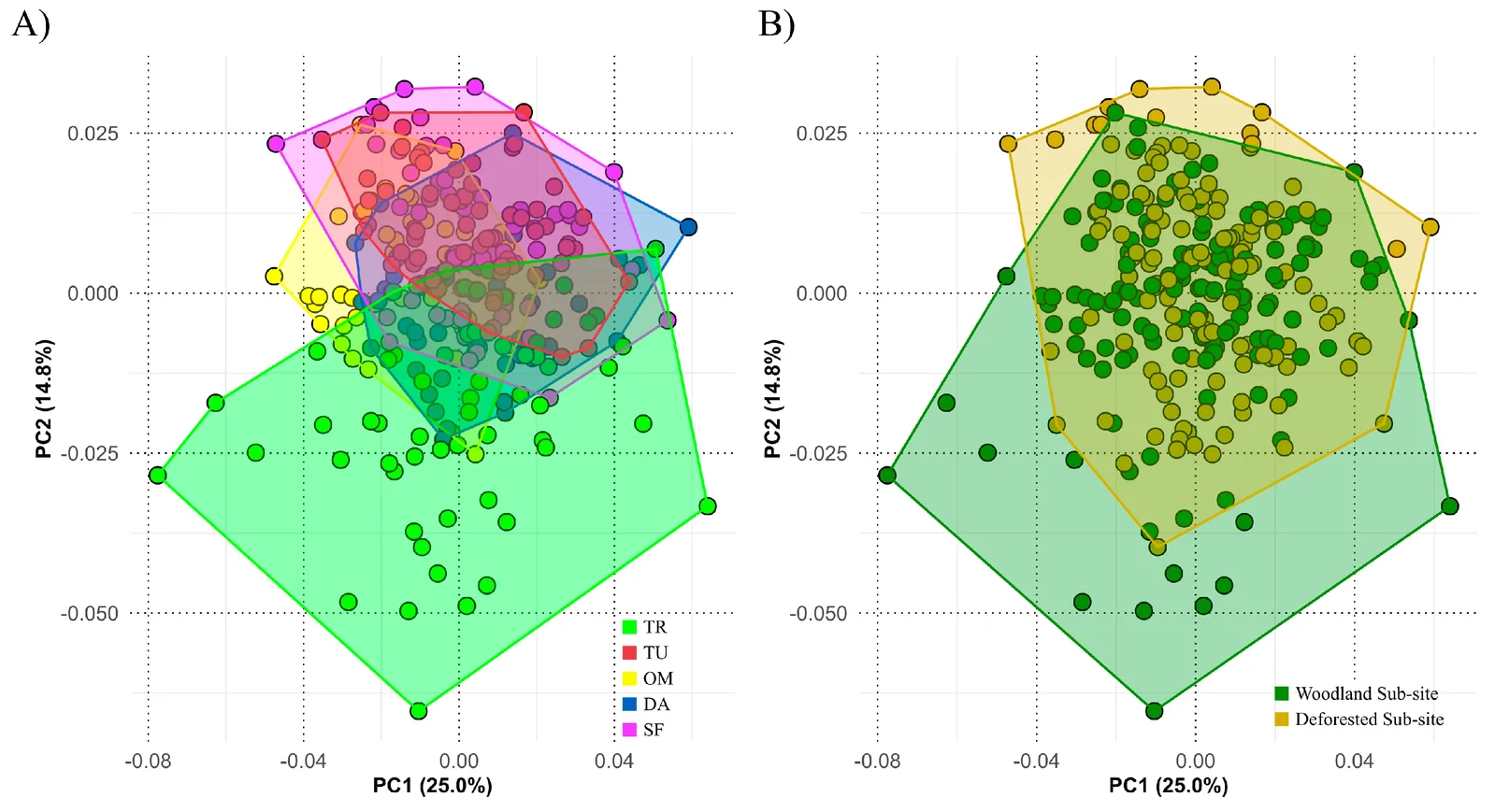
Principal Component Analysis (PCA) of right forewing shape in *Euglossa imperialis*. Points represent individuals and polygons indicate convex hulls: **A**) Morphological variation among sites: La Tronosa (TR, green), Túrega (TU, red), Omar Torrijos (OM, yellow), Darién (DA, blue), and Santa Fé (SF, purple). **B**) Morphological variation between woodland (green) and deforested (yellow) sub-sites. PC1 and PC2 explain 25.1% and 14.8% of total shape variation, respectively.

**Figure 4:**
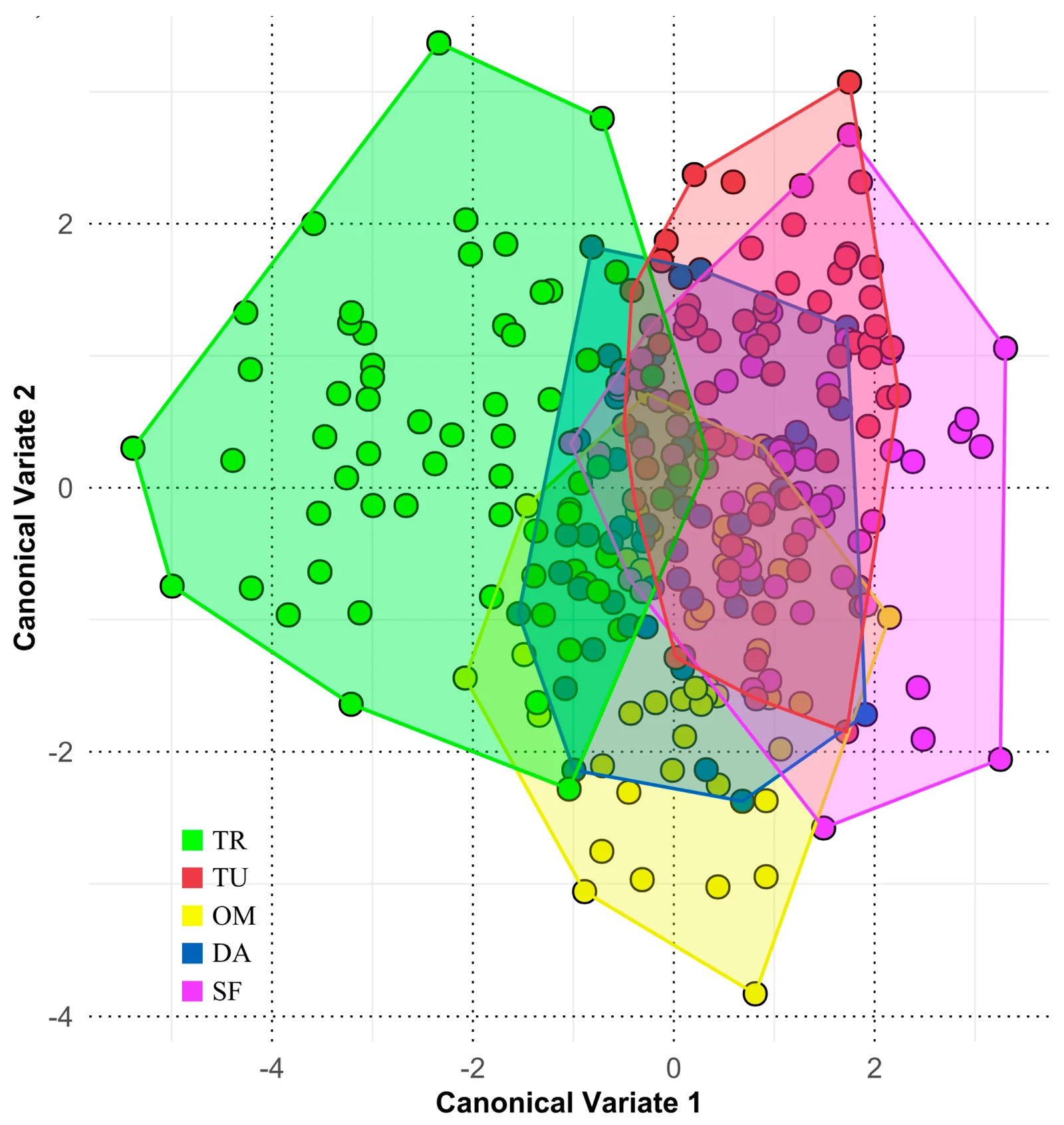
Canonical Variate Analysis (CVA) of right forewing shape in *Euglossa imperialis*. Points represent individuals and polygons indicate convex hulls. Wing shape variation among the five study sites: La Tronosa (TR, green), Túrega (TU, red), Omar Torrijos (OM, yellow), Darién (DA, blue), and Santa Fé (SF, purple).

Regarding morphology, the Principal Component Analysis (PCA) of the forewing shape shows that 39.9% of the variance is explained by the first two components (PC1 = 25.1%; PC2 = 14.8%). The morphospace of these two principal components grouped by sites (macro-scale; Figure 3A) shows a great overlap in shape among the four sites (TU, DA, SF and OM); however, the TR site presents a differentiation in shape from the other groups, with a higher variation in the morphospace across the first two PCs. In contrast, at a micro-scale level the morphospace shows a high overlap between shapes (Figure 3B), with a low differentiation between the sub-sites. Canonical Variate Analysis revealed marked morphological differentiation among the protected areas (Figure 4), with TR again showing the greatest separation from the remaining sites. Mahalanobis distances derived from the CVA, together with permutation tests, supported the same pattern observed in the PCA. Specifically, Tronosa exhibited the highest pairwise shape distances (Table 1), whereas the remaining sites showed lower levels of differentiation, consistent with their greater overlap in morphospace.

**Figure 5:**
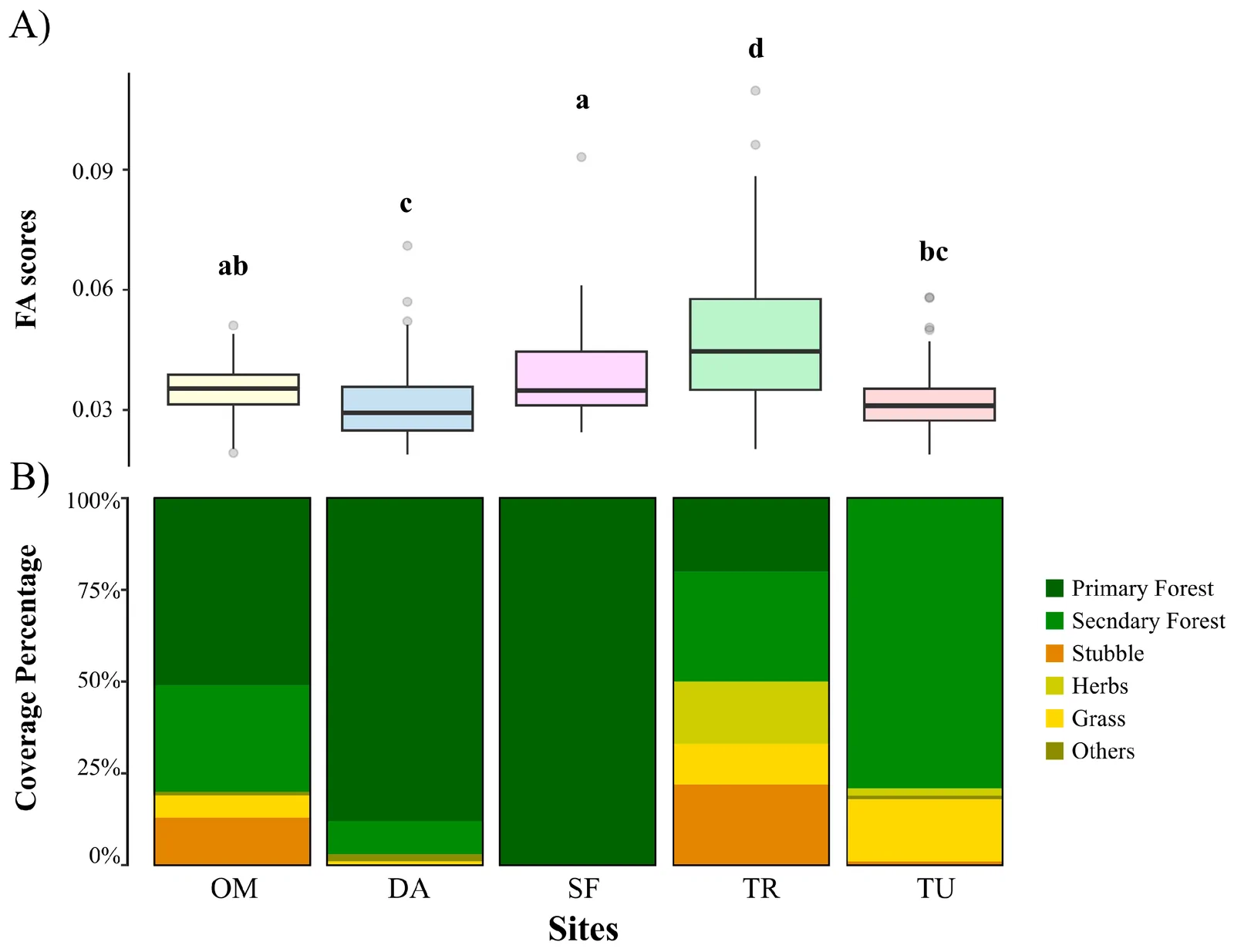
**A**) Fluctuating asymmetry (FA) scores across the five study sites. Significant differences were detected among sites (Kruskal–Wallis test, *p* < 0.05), with La Tronosa (TR) exhibiting the highest FA values and differing significantly from all the other locations. Letters indicate post hoc groupings. **B**) Land-cover composition of each site. The site with the highest deforested coverage (TR) also showed the greatest levels of FA, supporting a positive association between habitat disturbance and developmental instability.

On the other hand, significant fluctuating asymmetry values (FA) for each site were detected (*p* < 0.0001). The mean squares (MS) of ind*side (fluctuating asymmetry) exceeded the MS values for measurement errors (Table 2), suggesting that measurement error was negligible relative to biological variation in the landmark digitalization and that the observed FA could be attributed to non-methodological factors. In a macro-scale view, the Procrustes ANOVA also reveals different levels of FA (effect of the individual’s side on the asymmetric component of shape) among the sites (Figure 5A), ranking from highest to lowest FA as follows: (1) La Tronosa, (2) Santa Fé, (3) Omar Torrijos, (4) Darien, and (5) Turega. Interestingly, the TR site presents both the highest levels of FA (more than double that of the second site in the previous ranking) and deforested coverage (Figure 5A,B). Statistically, the results of the Kruskal–Wallis test show significant FA differences between Tronosa and the other sites (*p* < 0.05) (Figure 5A); however, despite the remaining sites presenting significant differences in FA between them, all are grouped together and are statistically different from TR (Figure 5A).

**Figure 6:**
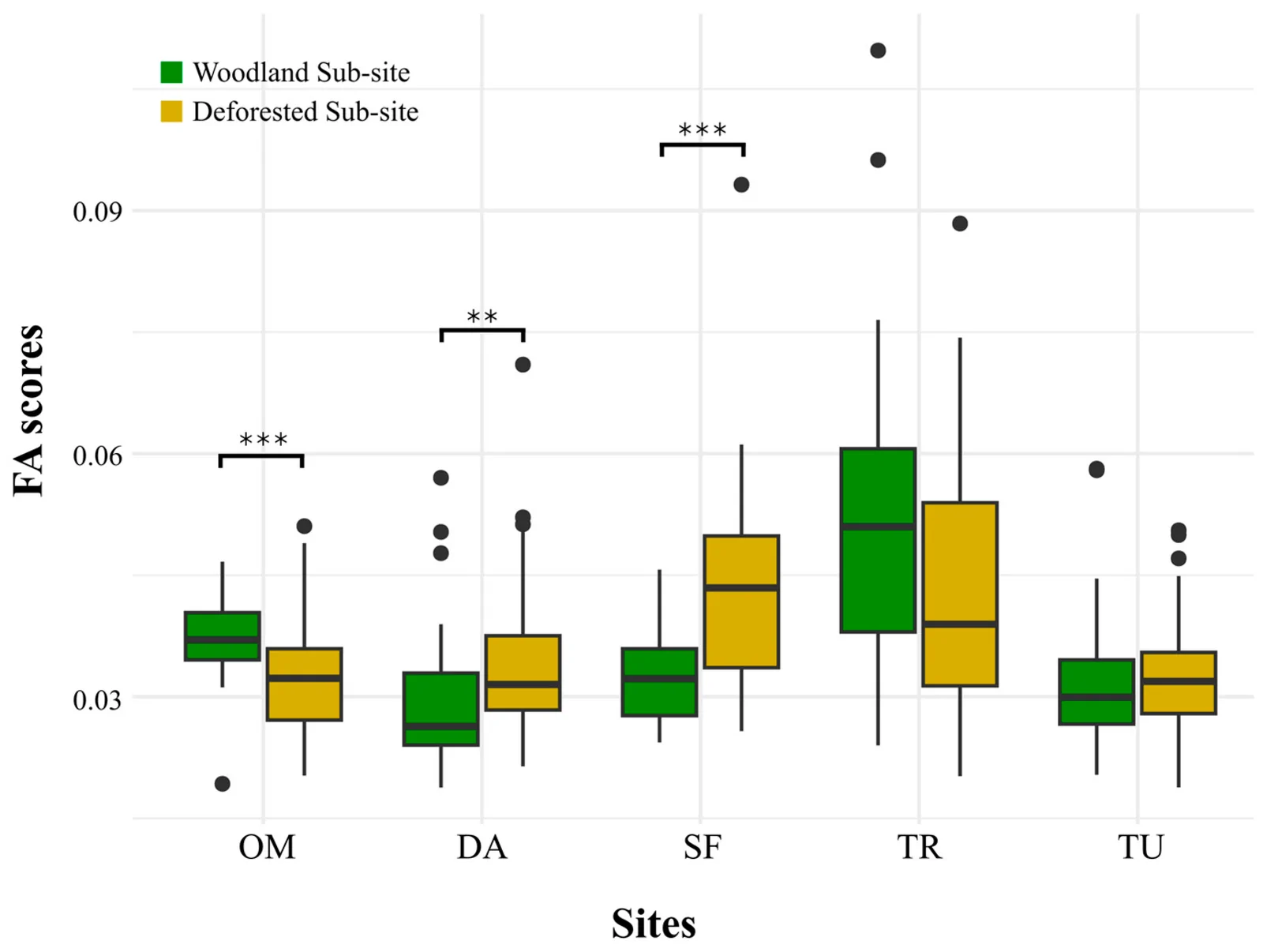
Comparison of fluctuating asymmetry (FA) scores between woodland (green) and deforested (yellow) sub-sites across five sampling locations. Significant differences between habitat types were detected only at OM, DA, and SF (Mann–Whitney U test, *p* < 0.05), while no differences were observed at TR and TU, indicating no consistent pattern of FA variation among sites. Significance levels are indicated as follows: ** = very significant (*p* < 0.01); *** = highly significant (*p* < 0.001).

The Mann–Whitney U tests show the lack of a discernible pattern; only three sites present significant differences in FA between the woodland and deforested sub-sites (*p* < 0.05; Figure 6). Both Santa Fé and Darien display higher levels of FA in the deforested sub-sites; however, OM shows the contrary pattern, with higher levels of FA in the woodland sub-site compared to the deforested sub-site. Finally, the generalized linear model results show a significant influence of sub-site and deforested coverage on the individual values of fluctuating asymmetry (Table 3; *p* < 0.001). In this analysis, both variables present a positive estimate, suggesting that at two scales (macro and micro) an increase in deforestation is associated with higher levels of FA; however, the deforested coverage estimate presents a higher value of this parameter, showing a greater influence of deforestation at the macro scale on the bees’ fluctuating asymmetry.

## 4. Discussion

This study provides strong evidence that landscape deforestation is associated with increased developmental instability in *Euglossa imperialis*. Among the five protected areas evaluated, La Tronosa exhibited both the highest proportion of deforested land and the highest levels of fluctuating asymmetry, while the sites with greater forest cover generally showed lower developmental instability. Furthermore, the positive relationship detected between deforested coverage and fluctuating asymmetry supports the hypothesis that habitat transformation imposes biological stress capable of disrupting normal developmental processes. These findings suggest that fluctuating asymmetry may represent a sensitive indicator of environmental degradation in orchid bees and provide new evidence supporting their use in conservation-monitoring programs.

The relationship between deforestation and fluctuating asymmetry observed in this study is consistent with the hypothesis that environmental disturbance disrupts developmental stability. Fluctuating asymmetry is widely recognized as the phenotypic expression of small random deviations arising during development when organisms are unable to fully buffer environmental or genetic perturbations, making it one of the most frequently used indicators of developmental instability [16,23,60,61]. Although fluctuating asymmetry has frequently been proposed as an indicator of environmental stress, empirical evidence in bees has been mixed. Positive associations have been reported under habitat disturbance, pollution, climatic stress, and nutritional limitation, whereas other studies have found weak or inconsistent relationships depending on species, traits, and environmental context [48,62,63,64,65,66]. Our results support the former pattern for *Euglossa imperialis*, suggesting that landscape-scale deforestation represents a sufficiently strong stressor to disrupt developmental stability in this species. These findings add to the growing body of evidence indicating that developmental instability can provide useful information on environmental degradation, although its value as a biomarker should be evaluated within the ecological context of each species. [45]. Within Euglossini, Silva et al. [67] reported higher asymmetry levels in populations exposed to climatic and anthropogenic influences, suggesting that developmental stability may be particularly sensitive to environmental disturbance in this group. Our results extend these findings by showing that landscape-scale deforestation is positively associated with wing asymmetry in *Euglossa imperialis*, supporting the use of developmental instability as an indicator of habitat degradation.

Several ecological mechanisms may explain the association between deforestation and increased developmental instability observed in *E. imperialis*. Forest loss alters habitat structure, reduces forest cover, and modifies the spatial distribution of resources available to orchid bees, potentially affecting both foraging behavior and population persistence. Orchid bees are strongly associated with forested environments, and studies have demonstrated that changes in forest cover and fragmentation can influence community composition, abundance patterns, and ecological interactions in Euglossini [68,69]. Recent evidence further indicates that forest cover is positively associated with pollen diet diversity and brood resource availability, suggesting that more forested landscapes may support nutritionally diverse and potentially more resilient populations [70]. Under this framework, the elevated fluctuating asymmetry observed in highly deforested landscapes may reflect developmental constraints associated with habitat degradation, reduced resource diversity, or increased environmental variability during development. Although the specific mechanisms underlying the observed asymmetry patterns were not directly evaluated in this study, our results are consistent with the hypothesis that habitat degradation compromises developmental stability and that these effects can be detected through wing asymmetry [45,67]. Future studies should explicitly evaluate the relative contribution of resource availability, nutritional stress, and microclimatic variation to better understand the pathways linking deforestation and developmental instability in orchid bees.

An important aspect of our results is that the relationship between developmental instability and habitat alteration was more evident at the landscape scale than at the local scale. While comparisons between the forested and deforested sub-sites within the protected areas did not consistently reveal significant differences in fluctuating asymmetry, the proportion of deforested land across the broader landscape was positively associated with developmental instability. This pattern is consistent with the ecology of orchid bees, which are highly mobile insects capable of exploiting resources over extensive spatial scales. Previous studies have shown that Euglossini responses to habitat structure and forest cover depend strongly on the spatial scale considered, with landscape-level variables often providing a better explanation of ecological patterns than local habitat characteristics [69]. Likewise, orchid bees are known for their remarkable flight capacity and their ability to move among forest patches across fragmented landscapes [68]. Consequently, individuals captured at a particular site may integrate environmental conditions experienced across a much larger area than the immediate sampling location.

Our findings therefore suggest that developmental instability in *E. imperialis* may be driven by cumulative landscape-level effects rather than by local habitat conditions alone. Interestingly, developmental instability appeared to be a more sensitive response to deforestation than overall wing morphology. Although some differences in wing shape were detected among the protected areas, shape variation did not show the same consistent association with forest cover observed for fluctuating asymmetry. This suggests that developmental instability and morphological differentiation may capture different biological dimensions of the response to environmental disturbance. Fluctuating asymmetry reflects an organism’s capacity to buffer developmental perturbations and may therefore respond rapidly to environmental stress, whereas changes in overall morphology may require stronger selective pressures or longer periods of environmental exposure to become evident. Similar distinctions between morphological variation and developmental instability have been discussed in previous studies, which found that environmental stress does not necessarily affect shape and asymmetry in the same manner [16,71]. In this context, our results suggest that fluctuating asymmetry may provide an earlier and more sensitive signal of habitat degradation than changes in wing morphology alone. Although this study demonstrates a clear association between landscape deforestation and developmental instability in *Euglossa imperialis*, some limitations should be acknowledged. We focused on landscape structure as the main environmental predictor, whereas other factors known to influence developmental stability, such as pesticide exposure, pathogen load, nutritional stress, and microclimatic variation, were not directly evaluated. Future studies integrating these variables will help clarify the mechanisms underlying the observed increase in fluctuating asymmetry and strengthen the use of orchid bees as bioindicators of environmental degradation.

Our findings contribute to the ongoing debate regarding the usefulness of orchid bees as indicators of environmental quality in tropical ecosystems. This debate has generated contrasting views, with some authors questioning whether orchid bees consistently fulfill the criteria required of reliable bioindicators [43], whereas others have highlighted their sensitivity to environmental change and their potential value for conservation monitoring [44]. While previous studies have often focused on species richness, abundance, or community composition as measures of habitat disturbance, our results suggest that developmental instability may provide an additional and potentially more sensitive indicator of environmental degradation. The positive association between landscape deforestation and fluctuating asymmetry indicates that individual-level responses can reveal ecological impacts that may not be fully captured through traditional community metrics alone. Because fluctuating asymmetry can be quantified using relatively simple morphometric approaches, it represents a promising tool for biomonitoring programs aimed at detecting the biological consequences of habitat loss and fragmentation. Collectively, these findings highlight the potential of developmental instability as an early-warning signal of environmental degradation and reinforce the value of integrating individual-level responses into biodiversity monitoring and conservation strategies in rapidly changing tropical landscapes. In this sense, our results provide additional support for the use of orchid bees as bioindicators of environmental quality, particularly when developmental instability is incorporated alongside traditional ecological metrics [44]. Although forest cover explained a significant proportion of the observed variation in developmental instability, other environmental stressors associated with habitat degradation, such as pesticide exposure, microclimatic alteration, or other anthropogenic disturbances, may covary with forest loss and also contribute to fluctuating asymmetry. Because these variables were not quantified in the present study, their independent effects cannot be separated and should be evaluated in future studies.

## Figures and Tables

**Table 1:** Mahalanobis shape distances between study sites extracted from Canonical Variate Analysis. Significant shape distances are shown in bold (*p* < 0.001). La Tronosa presents the highest distance, whereas Túrega shows the lowest values.

	OM	DA	SF	TR
DA	1.827			
SF	2.0721	1.7278		
TR	2.8401	2.6775	3.4007	
TU	2.3306	1.9079	1.5104	3.2669

**Table 2:** Results of Procrustes ANOVA assessing the effect on the asymmetric shape component of the individual (Ind), side (directional asymmetry), ind*side (fluctuating asymmetry), and site (macro-scale). Significant FA values and the effect of site on the asymmetric shape component are detected.

Effect	SS	MS	df	F	*p*
Ind	0.8253	0.0001129	7306	1.89	<0.001
Side	0.1967	0.0007566	26	12.63	<0.001
Ind*Side	0.4438	0.0000598	7410	2.46	<0.001
Site	0.2045	0.001966	104	17.41	<0.001
Error	0.3619	0.00002433	14,872		

**Table 3:** Generalized linear model results assessing the effects of sub-site (micro-scale) and deforested coverage (macro-scale) on fluctuating asymmetry (FA). Both variables showed significant positive effects (*p* < 0.001), indicating that higher levels of deforestation are associated with increased FA. The stronger effect of deforested coverage suggests a greater influence of landscape-scale deforestation on developmental instability.

Effect	Estimate	Standard Error	t-Value	*p*
Intercept	4.47	0.10	42.15	<0.001
Deforested Sub-site (micro-scale)	0.36	0.12	2.97	<0.001
Deforested Coverage (macro-scale)	1.75	0.34	5.16	<0.001

## Data Availability

Data is available and further inquiries can be directed to the authors.
